# The analysis for mitochondrial genome sequencing of *Triplophysa lewanggensis*

**DOI:** 10.1080/23802359.2016.1202740

**Published:** 2016-08-31

**Authors:** Huaiqing Deng, Huamei Wen, Ning Xiao, Jiang Zhou

**Affiliations:** a School of Life Sciences, Guizhou Normal University, Guiyang, Guizhou, China;; b Guiyang Nursing Vocational College, Guiyang, Guizhou, China

**Keywords:** Mitochondrial genome, phylogenetic tree, *Triplophysa lewanggensis*

## Abstract

*Triplophysa lewanggensis* revealed that the complete length of its mitochondrial genome was 16,568 bp, composed of A (31.7%), T (27.1%), G (15.9%), C (25.4%), and A + T (58.8%). Its genetic constitution and arrangement were consistent with other *Triplophysa*, including 13 protein-coding genes, 22 tRNA genes, 2 rRNA genes, and 1 control area (D-loop). All genes were encoded by the H-strand, except for one protein-coding gene (ND6) and eight tRNA genes (tRNA^Gln^, tRNA^Ala^, tRNA^Cys^, tRNA^Asn^, tRNA^Tyr^, tRNA^Ser(UCN)^, tRNA^Glu^, tRNA^Pro^) are encoded by the L chain, and the remaining genes are encoded by the H chain. The phylogenetic tree was divided into two main clades: one just including *Triplophysa* and the other including *Oreonectes*, *Homatula*, *Schistura*, *Nemacheilus*, *Traccatichthys. T. lewanggensis* is distributed in Southwest Guizhou Province attached to Yunnan–Guizhou Plateau, *T. xiangxiensis* is distributed in Hunan Province, they are near relatives, but their kinship with Triplophysa distributed in Tibetan Plateau is farther.

## Introduction

*Triplophysa* genera affiliated to Cobitidae family, Nemacheilinae subfamily, and it was known that there are about 100 species and subspecies in *Triplophgsa*, mainly distributed in the Tibetan plateau (Zhu 1989). *Triplophysa lewanggensis* is distributed in Southwest Guizhou Province attached to Yunnan–Guizhou Plateau. In the present study, the specimen was collected from Lewang town, Wangmo County of China (25°17.7873′ N, 106°18.7548′ E) in 2015. Now the specimen is deposited in the animal specimen room of the School of Life Sciences, Guizhou Normal University, Guiyang, China.

## Methods

Total DNA was extracted from the fish muscle tissues, and 26 pairs of primers were used to amplify genomic DNA. Then, complete mitochondrial genome was submitted to the GenBank (Accession no. KU987438).

Complete mitochondrial DNA sequences of 30 species of Nemacheilinae were obtained from GenBank, from which Cytochrome-b genes were extracted. The nucleic acids of cytochrome-b genes were then compared using the ClustalW method of MEGA version 6.0 software, the terminal irregular regions were removed manually. Subsequently, the phylogenetic tree was established using the neighbour-joining (NJ) method in MEGA 6.0, while reliability was tested using the Kimma2-Pamameter distance model and bootstrap method by repeating 1000 times.

## Results and discussion

The overall length of *T. lewanggensis* mitochondrial genome is 16,568 bp, including a total of 22 tRNA genes, two rRNA genes, 13 PCGs and a control region (D-loop). Twenty-eight genes are encoded on the H-strand, the remain nine genes including ND6 and eight tRNA genes (tRNA^Gln^, tRNA^Ala^, tRNA^Cys^, tRNA^Asn^, tRNA^Tyr^, tRNA^Ser(UCN)^, tRNA^Glu^, tRNA^Pro^) are encoded on the L-strand. The nucleotide sequence consists of A (31.7%), T(27. 1%), G (15.9%), C (25.4%), A + T (58.8%) shows a certain degree of A-T bias, the content of A + T in D-loop area was highest (68.8%).

**Figure 1.**
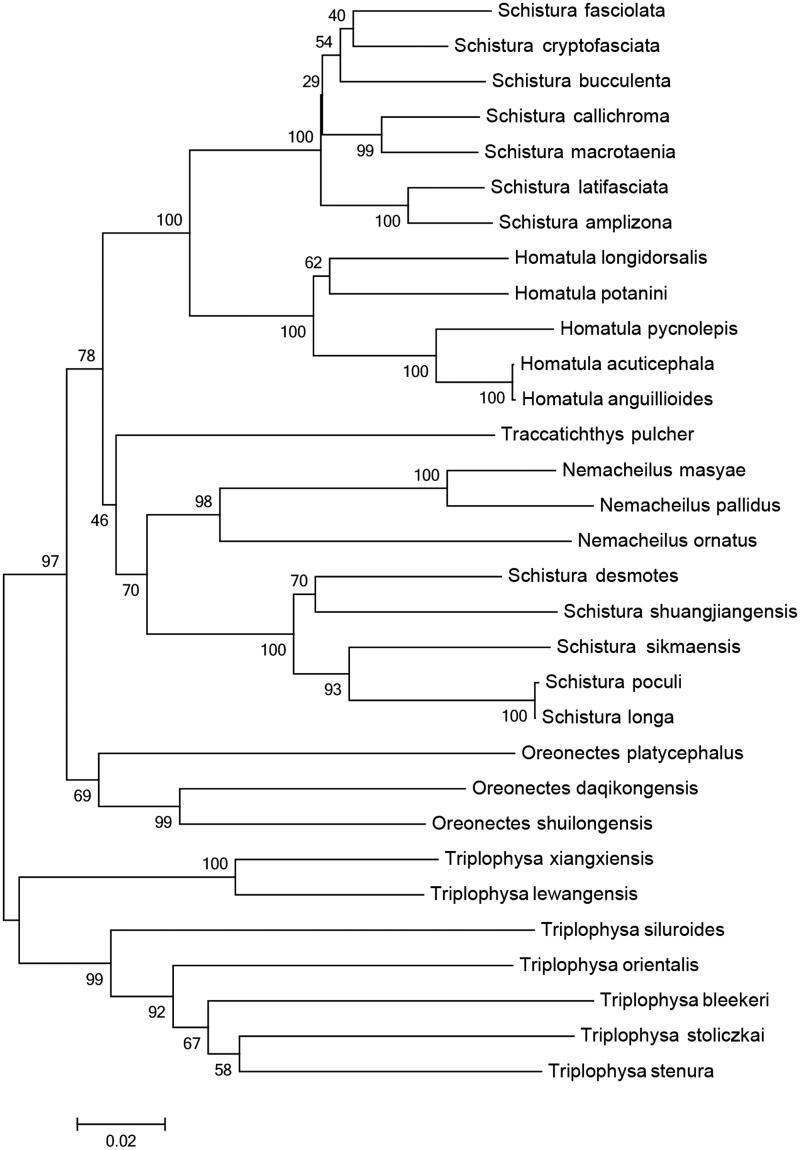
Inferred phylogenetic relationships among Nemacheilinae based on Cyt *b* gene sequence using NJ. Numbers at each node indicate percentages of bootstrap value (BP). Genbank accession number for the published sequences are *Oreonectes shuilongensis* (KF640641), *O. platycephalus* (DQ105197), *O. daqikongensis* (KU987436), *Schistura fasciolata* (HM010565), *S. shuangjiangensis* (JN837651), *S. desmotes* (GQ174368), *S. callichroma* (JN837652), *S. latifasciata* (JN837653), *S. bucculenta* (JN837654), *S. macrotaenia* (JN837655), *S. amplizona* (JN837656), *S. cryptofasciata* (JF340401), *S. sikmaiensis* (JF340405), *S. poculi* (JF340407), *S. longa* (JF340408), *Homatula pycnolepis* (KF041000), *H. acuticephala* (HM010527), *H. longidorsalis* (HM010550), *H. potanini* (JF340388), *H. anguillioides* (HM010582), *Traccatichthys pulcher* (JF340402), *T. xiangxiensis* (JN696407), *T. stoliczkae* (DQ105249), *T. siluroides* (EF212443), *T. bleekeri* (FJ406605), *T. stenura* (JN837657), *T. orientalis* (DQ105251), *T. lewangensis* (KU987438), *Nemacheilus maysae* (GQ174377), *N. ornatus* (GQ174363) and *N. pallidus* (GQ174370).

The phylogenetic tree was divided into two main clades: one just including *Triplophysa,* and the other including *Oreonectes*, *Homatula*, *Schistura*, *Nemacheilus*, *Traccatichthys* (Figure 1). The latter clade was also divided into two sister groups, the first one consisted of *Homatula*, *Schistura*, *Nemacheilus*, *Traccatichthys*, the other one only contained *Oreonectes*. *Triplophysa* divided into two branches, one branch is distributed in the high elevation area of the plateau containing seven *Triplophysa* fishes, each branch are clustering based on morphological classification relationship; another branch is distributed in the Yunnan–Guizhou Plateau containing two *Triplophysa* fishes. *T. lewanggensis* is distributed in Southwest Guizhou Province attached to Yunnan–Guizhou Plateau, *T. xiangxiensis* is distributed in Hunan Province, they are near relatives, but their kinship with *Triplophysa* distributed in Tibetan Plateau is farther.

Since mitochondrial genomic information for Triplophysa of Nemacheilinae is relatively rare, it is necessary to obtain mitochondrial genomes of more Triplophysa and other genus of Nemacheilinae in order to clarify the historical development of the Nemacheilinae. To further improve the reliability of the phylogenetic tree, a combination of information on the mitochondrial and nuclear genome is desirable to elucidate the phylogenetic relationships of the Nemacheilinae.
